# Factors that promote and sustain the use of traditional, complementary and integrative medicine services at LEKMA hospital, Ghana, 2017: an observational study

**DOI:** 10.1186/s12906-020-03185-y

**Published:** 2021-01-06

**Authors:** Angela Kenu, Ernest Kenu, Delia Akosua Bandoh, Moses Aikins

**Affiliations:** 1 School of Public Health, College of Health Sciences, University of Ghana, Legon, Accra, Ghana; 2 Ghana Field Epidemiology and Laboratory Training Program (GFELTP), Department of Epidemiology and Disease Control, School of Public Health, College of Health Sciences, University of Ghana, Legon, Accra, Ghana; 3 Health Economics, Systems and Policy Research Group (HESPRG), Department of Health Policy, Planning & Management, School of Public Health, College of Health Sciences, University of Ghana, Legon, Accra, Ghana

**Keywords:** Complementary and alternative medicine, Integrative health, Sustainability, Service providers, TCIM users, LEKMA hospital

## Abstract

**Background:**

About 70% of Ghanaians depend on traditional, complementary and integrative medicine (TCIM) practices for primary healthcare needs. It was therefore integrated into mainstream healthcare delivery system by the Ministry of Health in September 2012. LEKMA hospital was one of the institutions for piloting TCIM services. We assessed factors that promote the usage and sustainability of TCIM services within the formal healthcare system.

**Methods:**

We conducted a cross-sectional study from April–June 2017 at the LEKMA hospital, Accra, Ghana. Patients and managers of TCIM clinic were interviewed. Data was collected through qualitative and quantitative approaches. We defined usage of TCIM as its current use, and sustainability as structures in place to run TCIM services. For assessing usage, a five-point Likert scale was used to assess five domain areas via exit interviews. Managers were assessed on the sustainability of TCIM services through in-depth interviews. Likert scales responses were analysed quantitatively using descriptive tertile statistics. Thematic analysis was used for qualitative analysis.

**Results:**

Overall, 72.7% (40/55) of the clients showed a high preference for TCIM usage and 80.0% (4/5) of the managers valued it as partially sustainable. Eighty per cent (44/55) of patients indicated that the location of TCIM services and availability of visible directional signs influenced the good usage; 84% (46/55) of the patients agreed that the usage of TCIM was influenced by their perceived effectiveness. Managers indicated that human resources for providing services was a challenge and TCIM integration into the operations of the hospital needed to be improved.

**Conclusion:**

We observed a high preference for usage of TCIM among users at LEKMA hospital. The general belief in the potency, perceived effectiveness, location and availability of TCIM services are key determinants of the high preference for usage of TCIM. Provision of TCIM services in its current form is partially sustainable from the managers’ perspective. We recommend that the Ministry of Health ensures the availability of staff and create awareness of TCIM services among the general populace.

## Background

Traditional, complementary and integrative medicine and therapies (TCIM) refers to different forms of health practices and products outside the mainstream orthodox medicine [1]. TCIM is also referred to as complementary and integrative health. It covers a broad spectrum of ancient and modern approaches that support the prevention or treatment of diseases [2]. It includes the use of traditional herbs, traditional and spiritual healing, and other medical practices [2]. TCIM is known to be the oldest form of healthcare practice available and it is found in almost every country around the world [3].

Globally, the interest in TCIM and its use has grown and it is being accepted by many people around the world [4]. The WHO reports that about 80% of people in developing countries such as China, India, Latin America and large parts of Africa are known to rely on TCIM as a source of primary health care [5]. In Ghana, TCIM was practised over the centuries, and by trial and error, the knowledge of traditional medicine has been acquired and adopted [6]. It is known to have existed hundreds of years before the colonial era [7].

Colonization and activities of the missionaries in the country led to the introduction of conventional medicine [8]. This led to the repression of TCIM services in Ghana. Conventional medicine was therefore recognized and institutionalized as the mainstream healthcare in the country. However, TCIM has still been recognized by governments over the years as an existing healthcare system with rising patronage and usage.

About 70% of the population of Ghana depend on TCIM for their primary health care needs [9]. Ghana, therefore, implemented the WHO strategy of integrating TCIM into formal healthcare delivery system in September 2012 [6]. In 2011, traditional medicine practice was introduced into the mainstream health care system in 11 selected hospitals across the country. This strategy was to ensure that TCIM services are offered in a safe, respectful and effective manner according to the policies and regulations of the country [10, 11].

Studies have shown that sustainability and integration of TCIM depend on provider and client factors. These factors include financing, knowledge generation and management, capacity building, appropriate service delivery mechanisms, individual/community belief systems, and usage of TCIM [12–16]. This study assessed the usage and sustainability of integrating TCIM in a district hospital in Ghana from both the provider and patient perspectives.

**Fig. 1:**
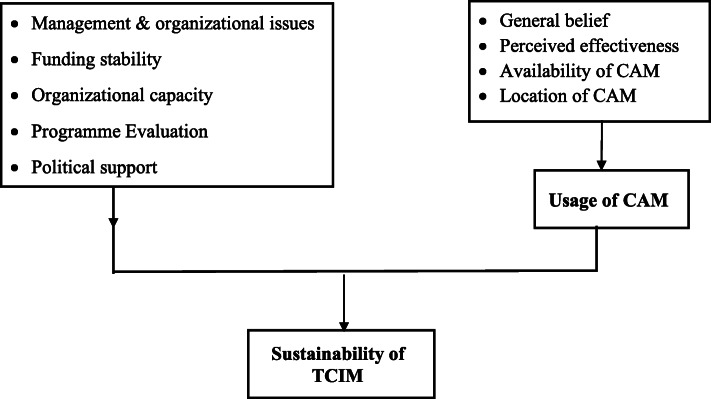
Conceptual framework of sustainability of traditional complementary and integrative medicines study

The conceptual framework of the study was based on usage by patients, leading to sustainability of the TCIM programme. Several patient factors affect the usage of TCIM services: general belief, perceived effectiveness, the availability and location which in turn creates demand. From the provider’s perspective, programme sustainability was assessed on its management and organizational issues, funding stability, organizational capacity, programme evaluation, political support, partnership and planning. These will ensure service availability and easy access to sustain TCIM services in the facility (Fig. 1).

## Methods

### Study setting

The study was conducted at Ledzokuku Krowo Municipal Assembly (LEKMA), a newly created district in the Greater Accra Region of Ghana. The LEKMA hospital is the major public hospital in the Ledzokuku Krowo municipality, providing healthcare to the majority of the inhabitants. It was built through a collaboration between Ghanaian and Chinese Governments in 2010. Before its construction, there was no public facility in the municipality. It is a 100-bed capacity hospital with 9 departments namely: Out-Patients Department (OPD); Maternity (Ante-natal and family planning); Dental; Eye; Laboratory; Ear- Nose -and Throat; Radiology; Dermatology and TCIM departments. The orthodox component of the hospital currently has 18 medical doctors among whom are: eight specialists, six medical officers and four house officers. There are 95 nurses, and 110 health extension/auxiliary workers. The average daily OPD attendance is 200 patients.

The hospital was one of the first hospitals in Ghana that integrated TCIM formally known as complementary and alternative medicine into its routine primary healthcare in 2011. The TCIM department provides complementary and alternative medicine therapy services such as; herbal medicine services, acupuncture, massage and chiropractic. At the Out-Patient-Department, patients decide to visit the TCIM facility to receive TCIM service based on their preference. The department is under the central management of the hospital. It has three trained medical herbalists, a nurse, a dispensing technologist, and a massage therapist. The attendance record at the TCIM department at LEKMA from January to May 2017 was 369 [17]. An average number of five patients visited the facility on a weekly basis.

### Study design and sampling technique

We carried out a cross-sectional study using mixed methods (quantitative and qualitative data collection methods) for data collection. Patients accessing TCIM services and some service providers at LEKMA hospital from April to June 2017 were interviewed.

Given the relatively small number of patients attending the TCIM clinic during the study period, all patients assessing TCIM services in the facility who consented to participate in the study were engaged in an exit interview. A total of 55 TCIM patients were interviewed. Five managers involved in TCIM services in the hospital were purposively selected and interviewed. These were: the medical director, nurse in-charge, and head of pharmacy, doctor-in-charge, and the pharmacy technologist at the TCIM department.

All patients above 18 years who assessed TCIM services in LEKMA hospital were eligible for the study.

### Pre-testing

Pretesting of the questionnaires were conducted at a Police Hospital in Accra. Police Hospital is among the hospitals that have recently also integrated herbal medicine into its healthcare system. The essence of the pretesting was to identify the appropriateness of the questions, to identify questions that needed adjustment before the main study.

### Data collection

Data collection was conducted from April to June 2017. The data were collected by a trained field assistant. A structured questionnaire was used for the patient exit interviews to assess the use of TCIM. The questionnaire covered data on socio-demographic characteristics, questions under the following domains; general belief in the use of TCIM, perceived effectiveness of TCIM, types of TCIM services, availability of TCIM services, and location of TCIM services to assess the use of TCIM. For managers, both in-depth interview guides were used. We collected data on the sustainability of TCIM services in the hospital focusing on stability of funding, partnership, planning, organizational capacity, evaluation of the programme, and political support. The in-depth interview guide also covered the components of sustainability such as infrastructure, human resource, threats, and opportunities for improving TCIM services. Interviews were conducted in vacant consulting rooms or offices on the wards with no interruptions. To ensure that all details of responses were captured, permission was obtained for the digital audio recording of interviews to complement notetaking. These interviews lasted approximately 30–45 min.

### Data entry and management

Completed questionnaires were reviewed for completeness. To ensure reliable data entry, the data were double entered by two independent data entry clerks. Both entries were then generated and checked to further ensure that all errors are corrected. The digital audio-recorded interviews were transcribed verbatim. Both quantitative and qualitative data sets were password-word protected.

### Definition of terms

We defined TCIM usage as current use of TCIM services in the facility based on the patient’s general belief in it and their perspective of its effectiveness, availability and convenience of location. We defined sustainability as structures in place which enable TCIM services to run on its own with minimal support.

### Measurements and data analysis (statistics)

#### Usage assessment

Patient usage of TCIM was measured and analysed using a 5-point Likert scale to assess the set of statements in the domains of the general belief in the use of TCIM, perceived the effectiveness of TCIM, types of TCIM, location of TCIM and availability of TCIM services. We used 5 dimensions of rating for the domain statements: Strongly disagree = 1; Disagree = 2; Neutral = 3; Agree = 4 and Strongly agree = 5. Each set of domain statements were rated. The responses and their respective scores for the 5 domain statements were determined for each patient. This was then used to describe the usage of TCIM services. Using descriptive tertile statistics, the total scores of TCIM usage were classified into 3 composite usage scores viz.: low (15–35); moderate (36–56); and high (57–75).

#### Provider sustainability assessment

Provider sustainability of TCIM services was measured and analysed using a 5-point Likert scale to assess the following domains: management and organizational issues, funding stability, organizational capacity, programme evaluation, political support, partnership, planning. The 5 dimensions of rating the domain statements were: Strongly disagree = 1; Disagree = 2; Neutral = 3; Agree = 4, and Strongly agree = 5. These were then used to describe the sustainability of TCIM services. Using descriptive tertile statistics, the total scores of TCIM sustainability was then used to develop 3 composite TCIM sustainability scores namely: low (19–32), moderate (33–46), and high (47–57) sustainability. Furthermore, thematic content analysis was carried out to capture relevant themes and patterns relating to sustainability from the in-depth interviews with the aid of NVIVO 7 qualitative data analysis software. Thematic content analysis was used to identify the major themes. This gave us a deeper understanding of the use and usage of TCIM services in the facility.

### Ethical considerations

Ethical approval for the study was granted by the Ghana Health Service Ethics Review Committee in March 2017 (Protocol Number GHS-ERC: 125/02/17). Permission was sought from the Greater Accra Regional and District Directors of Health Services, and the management team of LEKMA Hospital. The purpose and scope of the study were explained to eligible patients. Only those who consented to take part in the study were interviewed.

## Results

### Background characteristics of mangers

A total of 5 service managers were interviewed, 3 females and 2 males. Three of them were directly involved in the day to day running of the herbal unit. Three had worked for more than 10 years and 2 had worked less than 10 years.

### Background characteristics of patients

A total of 55 out of 60 patients participated in the study. The 55 patients were made up of 70.9% (39/55) females. The patients’ ages were between 18 to 85 years, with a mean age of 53.9 years (SD: 15.6 years). Table 1 shows the demographic characteristics of patients in the exit interview.

**Table 1:** Demographic characteristics of patients accessing TCIM unit of LEKMA Hospital, 2017

Variable	Number (%)
**Age**	
< 29	5 (9.1)
30–49	14 (25.5)
50–69	27 (49.1)
≥ 70	9 (16.3)
**Sex**	
Male	16 (29.1)
Female	39 (70.9)
**Educational level**	
Primary	7 (12.7)
Secondary	27 (49.1)
Tertiary	11 (20.0)
Non-formal	10 (18.2)
**Marital status**	
Married	28 (50.9)
Not married	27 (49.1)
**Religion**	
Christian	45 (81.8)
Muslim	7 (12.7)
Others	3 (5.5)
**Ethnic group**	
Ga	23 (41.8)
Akan	15 (27.3)
Ewe	11 (20.0)
Dagbon	6 (10.9)
**Occupation**	
Trader	13 (23.6)
Artisan	12 (21.8)
Professional	10 (18.2)
Unemployed	20 (36.4)
Total	55 (100.00)

### Usage of traditional, complementary and integrative medicine (TCIM)

About 73% (40/55) of responding patients indicated high usage of TCIM whilst the remaining 27.2% indicated low usage. Patients believed that the more knowledge one has on TCIM the more likely one will use it. About 76% (42/55) of patients agreed that the location of TCIM services influenced their decision to use the service. Concerning the perceived effectiveness of TCIM, 61.8% (34/55) of patients agreed that TCIM is more effective than orthodox medicine for the treatment of their condition. On the usage of TCIM, 85.5% (47/55) of patients indicated that they will recommend TCIM to their friends and family (Table 2).

**Table 2:** Patient’s views on factors that promote TCIM usage, LEKMA hospital, 2017

Client issues	Disagree (%)	Neutral (%)	Agree (%)
**General Belief in the use of** TCIM			
Knowledge in the use of TCIM	3 (5.4)	9 (16.4)	43 (78.2)
More potent than orthodox medicine	5 (9.1)	14 (25.5)	36 (65.5)
Can be dangerous	8 (9.6)	6 (10.9)	41 (74.6)
Last resort	19 (34.5)	8 (14.6)	28 (50.9)
**Location of TCIM**			
Location influences decision	9 (16.4)	4 (7.3)	42 (76.4)
More people will use if located in a health facility	3 (5.5)	2 (3.6)	50 (90.9)
Visible directional signs	15 (27.3)	6 (10.9)	34 (61.9)
**Perceived effectiveness**			
More effective	6 (10.9)	15 (27.3)	34 (61.8)
Decide before coming	7 (12.7)	1 (1.8)	47 (85.4)
**Availability of TCIM services**			
Readily available	8 (14.5)	13 (23.6)	34 (61.8)
Waiting time	5 (9.1)	10 (18.2)	40 (72.7)
Cost is cheaper	23 (41.8)	17 (30.9)	15 (27.3)
Included in NHIS	2 (3.6)	3 (5.5)	50 (90.9)
TCIM service is easier	15 (27.3)	14 (25.5)	26 (47.3)
**Perceived effectiveness**	**Definitely no**	**Not sure**	**Definitely yes**
Recommendation of TCIM to family and friends	4 (7.2)	4 (7.23)	47 (85.5)

### Mangers’ perspectives of sustainability of TCIM services

**Fig. 2:**
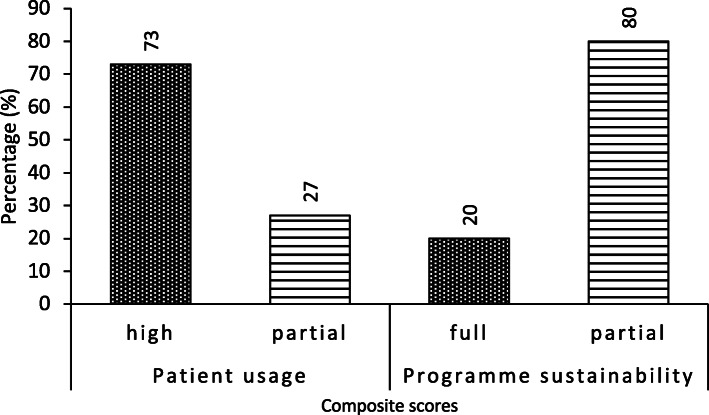
Composite scores of usage and sustainability of traditional, complementary and integrative medicine, LEKMA hospital, 2017

Four (4) of the five (5) responding managers indicated that sustainability in the hospital was partial (Fig. 2). Managers supported their assertion of partial sustainability of TCIM with the following observations.

*“It’s in its growing stages. It’s quite new in Ghana. Before, TCIM used to be only for foreigners who have come in but now locals are getting to know TCIM and it’s beginning to spread. In the past, if you wanted TCIM services, probably you will need to go to a Chinese clinic or some foreigner’s place. But now we are finding them in even private clinics owned by Ghanaians.*”(Respondent 2)

“C*urrently, what we are doing is just one part of TCIM, and not the whole service just because people do not like the medications, the herbal part seems to be dying, whiles the massage and other… the acupuncture seems to be growing. But if we are looking for a holistic service, all these services must go together.”* (Respondent 1)

Managers’ assessment of TCIM sustainability was on funding, organizational capacity, programme evaluation, political support, partnership and planning. Underfunding; managers said the TCIM budget was part of the LEKMA hospital budget but inadequate. All the managers agreed that salaries were paid by the government. However, TCIM has not gotten sustained funding from the government to date.

On the issue of the current state of TCIM in LEKMA, a manager indicated that:

*“The present, it is not good. We have a lot to do. The past was better because we were distributing flyers, we were talking to patients, we were making announcements downstairs for people to know of the herbal services upstairs, we were talking to the doctors so they can refer patients to us. But such things have ceased because the microphone over there is spoilt”* (Respondent 3)

On the issue of infrastructure to sustain the service, the responses were that there were not enough to sustain the service:

*“When LEKMA was built, there was a whole sector for the herbal division, the Chinese wanted to do a lot because they wanted to bring in some machines and other equipment, but before long, the rooms were given out. Now we have just two single rooms. The infrastructure is not enough. We still need rooms for so many things.”* (Respondent 3).

“*Personally, everything we are using is donated. So, when the donations stop coming in then, it will become a bit difficult”*. (Respondent 2)

They all mentioned that to sustain, they need to improve on the service

*“Okay, so there is this issue of low client turn over because people are not even aware this service is available, sometimes it seems like we are not being very useful”.* (Respondent 2)

*‘I said earlier on a lot more could be done… we are not supposed to advertise too’* (Respondent 3)

Whereas four managers agreed that TCIM’s integration into the operations of the hospital was little, the fifth manager asserted that TCIM had been integrated to a large extent. Only one manager thought there was no organizational system in place to support its various needs.

Of the five (5) managers, three (3) were of the view that there was evidence showing the useful services the unit was providing and that the services of the unit were being monitored. All managers agreed that the unit submitted regular reports.

Human resources was an issue on the sustainability of the services. The managers thought that this too was a limitation to service provision …

“Some personnel are not permanent. So, they just come in for a specific time and then they have to go away. If this continues, it will not be something we can sustain” (Respondent 2)*.*

The doctor in charge remarked that: “the TCIM services are currently sustained by the Chinese support, should they withdraw this support at any time, the system is very likely to collapse”

They also mentioned that a staff is currently being trained in China.

“One of our ladies is currently in China, learning something different from what she was trained to do, she is a herbalist, but now learning acupuncture, this is also to help with sustainability” (Respondent 1)

The main threats to sustainability pointed was the shortage of drugs and lack of variety of drugs at the pharmacy.

“There is a list of herbal medicines that government has put in place. Those are the approved drugs that we are to use. We can only use drugs from Mampong. That is one of the problems. Sustainability means that we should stock a variety of drugs at all times “ (Herbal Technologist)

“ Sometimes we do run short of some of the vital drugs at the pharmacy, because of the way they are purchased”. (Respondent 5)

“In my opinion, like the private pharmacy, we should also have the essential drugs in stock every time, so clients do not have to leave the hospital to go and purchase drugs from outside” (Respondent 5)

All the patients agreed that Ghana Health Service garners resources for TCIM services. Three (3) out of the five (5) managers agreed that the unit had political support from local authorities. Secondly, 3 out of 5 of mangers also said Ghana Health Services advocates for TCIM services.

Two out of five managers agreed that TCIM had public support to a little extent and community and stakeholder support to a large extent. Three out of five managers were also of the view that local leaders such as community leaders should be engaged in the development of TCIM goals to some extent.

Only one manager was of the view that the LEKMA Health Directorate was to a large extent involved with plans of TCIM operation and expansion; although three of the managers said the plans of the unit were integrated into the main plan of the hospital to a large extent and run solely by the hospital.

Another respondent said the slow effect of herbal treatment is a threat which could discourage clients if the service providers are not confident enough to convince them of its effectiveness. Also, there is a problem with low client usage, they all mentioned the lack of advertisement as a threat to the service.

“People want the accuracy of what you are giving to them, they want to make sure it will work, herbal is slow but it’s very effective.” (Respondent 3)

‘Ghana Health Service does not permit us to advertise, so how are they helping people to know what we do?” (Respondent 3)

“If we were able to advertise, ‘YES’ that will be a great opportunity.” (Respondent 2)

## Discussion

We assessed the usage and sustainability of these services at LEKMA hospital, a public facility at the district level. From the clients’ perspective, the high usage of TCIM among users of TCIM at LEKMA indicated that it was sustainable. However, from the health service providers’ perspective, it was partially sustainable.

From this study 75% of the patients indicated high usage of TCIM. This result was similar to findings by James et, al. which indicated that utilization of TCIM in recent years has grown dramatically [18]. Previous studies indicated that high usage of TCIM is linked with its low cost, accessibility and affordability [2, 16].

On general belief, the majority of the patients believed that TCIM was more potent than orthodox medicine and that it was able to treat their condition better than orthodox medicine. Findings from this work support an earlier work by Gyasi and his colleagues who reported that people believe TCIM was potent, powerful, and very useful in the treatment of various ailments [13]. Other people find the use of TCIM to be physiologically comfortable because they believe this system of health was embedded in their socio-cultural traditions [13]. About half of patients believe that TCIM should be used as a last resort and this corroborates findings by Verhoef and colleagues, in their study which indicated that TCIM can be used as a last resort and finding hope [19].

This study found several factors that influence people’s decision to use TCIM, these are the availability of visible directional signs to direct people and the fact that TCIM was located in the health facility. This may be explained by the fact that once it was located within the premise of the health facility it means it has been recognized as part of the health system and users may feel comfortable that their activities will be regulated.

Patients who responded to the study perceive TCIM to be very effective for the treatment of their conditions. A study in Ghana on perceptions and use of TCIM among pregnant women in Ghana also recorded positive perceptions on the effectiveness of the use of TCIM [20].. A study in the Caribbean revealed that traditional medicines were perceived to be more efficacious in some instances or as equally effective as orthodox medicines [21]. This assertion was similar to the findings in this study. Earlier work done in the Ashanti region found that clients perceived traditional medicine to be effective. This was supported by their experiences of healing from conditions such as hypertension, boils, broken bones/fractures, impotency and infertility etc. Some even believed as far as these diseases were concerned, it was more effective than conventional medicine [13]. A study by Muftawu, on integration in the same hospital, observed that herbal medicine was in high demand due to the increase in chronic non-communicable diseases [22].

The availability of medications and logistics such as wards, improved drug list and more personnel for treatment largely influence the use of TCIM whereas the absence of the logistics invariably means even those who depend on TCIM fully will not be able to use it. The availability of these needed logistics to effective use of TCIM cannot be over-emphasized. This was also linked to the sustainability of TCIM services.

The study revealed that complementary and alternative medicine at LEKMA hospital was partially sustainable because of the inadequate human and material resources. A critical assessment of the components of sustainability clearly shows that there was the need to pay much attention to the availability of human resource, availability of requisite infrastructure, adequate funding, organizational capacity, programme evaluation, political support and partnership. In addition, funding of TCIM was under threat since there was little or no sustained funding from the government. Ensuring that TCIM services are absorbed by the national health insurance scheme (NHIS) should be considered. The unavailability of government resources for the acquisition of laboratory equipment to conduct further experiments and the cultivate medicinal plants to improve existing herbal products in the country and to come up with safe, effective and quality herbal products weakens this arm of service delivery [23]. With available old laboratories and facilities, institutions such as LEKMA should take advantage and rehabilitate them and use them to help improve traditional medicine development in the country. KNUST trains traditional medicine practitioners with formal knowledge of medicinal plants. These graduates apply scientifically-harnessed herbal medicines for the treatment of diagnosed diseases using locally available resources [24]. Again, the Traditional Medicine Practice Council contribute to building the capacity of its members by rolling out a series of capacity building training and continuous professional development programmes [24]. Managers commended the human resource capacity the Chinese government contribute towards running of the clinic.

The organizational system described by the managers falls short of what Essegbey & Awuni, defined as an important first step in the integration of traditional medicine into the healthcare system of the country which includes oversight responsibility from Traditional and Alternate Medicines Directorate that was set up under the Ministry of Health [25]. The addition of some herbal medicines to the Essential Drug List and ensuring some traditional medicine treatments are covered by the NHIS have all not been implemented.

There was demonstrable evidence of the usefulness of the TCIM according to the managers. In addition to some level of monitoring of the services, there was a regular report generated on service provision. Documentation of service provision is only worth the effort if it is disseminated to the wider community to inform, educate, and produce desirable attitudes towards regulated TCIM use as practised in LEKMA and other accredited service centres.

The policy support for the integration of TCIM services into the mainstream healthcare system may be described as waning because logistics needed to conduct advocacy for TCIM has been dwindling over the years. Per the policy of GHS, advertisement on TCIM is not expected but there is the need to showcase the range of TCIM services being provided alongside the orthodox. Besides, the frequent shortage of drugs, non-availability of variety of products threatens the sustainability of TCIM.

Government of Ghana partnership with the Chinese Government has yielded a lot of benefits including the infrastructure for TCIM treatment, regular support with equipment and human resource. Local leaders’ engagement has been ongoing but their presence is not felt beyond period of durbars. However, they have been instrumental in providing the peaceful environment for TCIM operations at LEKMA.

It was evident that the plans of the unit have been partially integrated into the main plans of the hospital. It was the same managers who oversee both the orthodox and TCIM facility and they ensure its functionality.

From our assessment, the high preference for usage depicts the willingness of patients to assess TCIM services if they are made available in government facilities. However, the structures in place may currently not be able to sustain the service. There is therefore the need for government and other stakeholders to revisit the existing structures to create an enabling environment to ensure that TCIM services provided in the facility are fully sustainable.

This study was an assessment of the feasibility of TCIM services into convectional medicine settings carried out for policy decisions. Therefore, it a basic descriptive approach with simple statistics was used for easy understanding. However, since LEKMA is the first and main Ghana Health Service facility which offers this service, the TCIM service of the hospital is a reflection of TCIM in public health services in Ghana. Again, conventional healthcare providers and managers of other conventional medicine departments were not interviewed about TCIM sustainability and use in the facility.

## Conclusion

Among the users of TCIM, the assessment of TCIM at LEKMA hospital revealed a high preference for usage of TCIM from clients’ perspective and partially sustainability from health service providers’ perspective. Usage of TCIM from clients’ perspective indicated that sustainability was due to their high general belief in the potency, knowledge in its use and serve as a last resort when all else fail. Other factors that positively influence the high usage of TCIM among the users include the location, perceived effectiveness and availability. Unfortunately, the study showed that the current state of affairs for TCIM at LEKMA may not be sustained from the service provider’s perspective. TCIM services can be sustained at LEKMA provided Government of Ghana through Ministry of Health and the Ghana Health service, ensures availability of staff, advocacy of the service to be covered by the National Health Insurance and education and awareness creation on the TCIM service in the hospital. We would like to recommend that further studies in the sustainability of TCIM services from the perspective of all service providers and other patients assessing other services in the facility carried out. This would help give a better insight into TCIM usage.

## Supplementary Information

**Additional file 1.** Dataset for journal revised. Responses of patients assessing TCIM services in the facility.

## Data Availability

The raw data is available upon reasonable request.

## Abbreviations

TCIM: Traditional, complementary and Integrative Medicine
GHS: Ghana Health Services
KNUST: Kwame Nkrumah University of Science and Technology
NHIS: National Health Insurance Scheme
OPD: Out Patient Department
SD: Standard Deviation
